# The use of essential oils as a growth promoter for small ruminants: a systematic review and meta-analysis

**DOI:** 10.12688/f1000research.24123.2

**Published:** 2020-08-28

**Authors:** Faizal Andri, Asri Nurul Huda, Marjuki Marjuki

**Affiliations:** 1 Doctoral Program of Animal Science, Faculty of Animal Science, Gadjah Mada University, Yogyakarta, 55281, Indonesia; 2 Department of Animal Nutrition and Feed Sciences, Faculty of Animal Science, Brawijaya University, Malang, 65145, Indonesia

**Keywords:** Antibiotics alternative, Average daily gain, Goats, Natural feed additives, Protozoa, Secondary metabolites, Sheep.

## Abstract

**Background:** Due to their antimicrobial properties and safety, essential oils are currently proposed as a sustainable option for antibiotic alternatives in the livestock sector. This current systematic review and meta-analysis investigated the effects of dietary essential oil supplements on dry matter intake (DMI), average daily gain (ADG), and feed conversion ratio (FCR) of small ruminants.

**Methods:** A total of 12 studies (338 small ruminants) were included in this meta-analysis. The overall effect size was quantified using Hedges’ *g* with 95% confidence interval (CI) using a fixed-effect model. Publication bias was inspected using Begg’s and Egger’s tests, followed by trim and fill method to detect the number of potential missing studies.

**Results:** Insignificant heterogeneity among studies was detected both on DMI ( *P* of Q = 0.810; I-square = 0.00%), ADG ( *P* of Q = 0.286; I-square = 17.61%), and FCR ( *P* of Q = 0.650; I-square = 0.00%). The overall effect size showed that essential oils supplementation had no significant impact on DMI (Hedges’ *g* = -0.12; 95% CI = -0.50 to 0.26; *P* = 0.429) and FCR (Hedges’ *g* = -0.17; 95% CI = -0.55 to 0.22; *P* = 0.284), but had a significant positive impact on ADG (Hedges’ *g* = 0.44; 95% CI = 0.12 to 0.76; *P* = 0.002). The result of publication bias analysis showed that DMI, ADG, and FCR did not present any significant biases ( *P* > 0.10), and no potential missing studies detected.

**Conclusions:** Dietary essential oil could improve ADG of small ruminants, without any alteration on DMI and FCR. Further research in this topic is still required to provide stronger evidence of the potency of essential oil as a growth promoter for small ruminants.

## Introduction

In animal nutrition, antibiotics become the first choice of feed additive due to their substantial benefit toward health and productivity. However, the routine use of this chemical additive yields residues in livestock products, and is also responsible for the development of microbial antibiotic resistance ^1, 2^. These factors represent a dangerous risk to human health, which has led to the global drive to reduce antibiotic use in the livestock sector. As a result, several natural products have been proposed to be used as antibiotic alternatives ^3, 4^.

Among natural feed additives, essential oils have a unique mechanism of action in livestock production. They can manipulate rumen fermentation characteristics ^5, 6^ and subsequently improve growth rate ^7, 8^. However, other findings showed no meaningful effect of this feed additive on productive performance ^9, 10^, while another study showed a negative impact ^11^. The inconsistent results among studies requires an appropriate tool to quantify the overall effect. Therefore, this study was conducted to measure the quantitative effects of dietary essential oil supplementation on dry matter intake (DMI), average daily gain (ADG), and feed conversion ratio (FCR) of small ruminants using a systematic review and meta-analysis approach.

## Methods

The systematic review and meta-analysis was conducted according to the Preferred Reporting Items for Systematic Reviews and Meta-Analyses (PRISMA) guideline ^12^. A completed PRISMA checklist is available in *Reporting guidelines* ^13^.

### Eligibility criteria

The inclusion and exclusion of the study were based on participants, interventions, comparisons, outcomes, and study design (PICOS) criteria as indicated in Table 1. Additionally, only publications written in English which was included in this study. All dates up until the date last searched were included.

**Table 1.** PICOS criteria.

	Inclusion criteria	Exclusion criteria
Participants	Sheep or goat	Other species
Interventions	Dietary essential oil supplementation	Irrelevant treatment
Comparisons	Control group (basal diet only)	
Outcomes	DMI, ADG, and FCR	No related outcome
Study design	Randomized controlled *in vivo* trials	*In vitro* trials

DMI: dry matter intake; ADG: average daily gain; FCR: feed conversion ratio.

### Literature search strategy

The literature search was carried out using the following electronic databases: Scopus, PubMed, and SciELO. The search was last performed on 30 April 2020. Table 2 shows the full electronic search strategy.

**Table 2.** Search strategy.

Database	Search strategy
Scopus	(TITLE (oil) AND TITLE (growth OR performance) AND TITLE (sheep OR goat OR lamb OR kid))
PubMed	((oil[Title]) AND (growth[Title] OR performance[Title])) AND (sheep[Title] OR goat[Title] OR lamb[Title] OR kid[Title])
SciELO	(ti:(oil)) AND (ti:(growth OR performance)) AND (ti:(sheep OR goat OR lamb OR kid))

### Study selection

Results from the search were firstly checked for duplicates. After duplicate studies were removed, the titles and abstracts were screened using the eligibility criteria ( Table 1). Full texts of the selected studies were then further examined to find eligible studies. The authors of the included studies were not contacted for further clarification.

### Data extraction

Data extracted included the following items: 1) authors; 2) animal species; 3) number of animals; 4) type of ration; 5) essential oil source; 6) experimental design, and 7) growth response variables. Growth response variables consisted of DMI, ADG, and FCR. Standard error or standard error of means were converted into standard deviation ^14^. The data was pooled when a study used more than one dose of essential oils or tested both sexes of experimental animals ^15^.

### Effect size quantification

The overall effect size was quantified using Hedges’ *g* ^16^ using a fixed-effect model. This model was chosen due to the insignificant heterogeneity among studies after checked using Cochran’s Q ^16^ and I-square ^17^.

### Publication bias analysis

Publication bias was inspected using Begg’s ^18^ and Egger’s tests ^19^, with *P* <0.10 set to determine the existence of publication bias. The trim and fill method ^20^ was employed to detect the number of potential missing studies and to adjust the overall effect size. All meta-analysis procedures were performed using Meta-Essentials version 1.4 ^21^.

## Results

**Figure 1.**
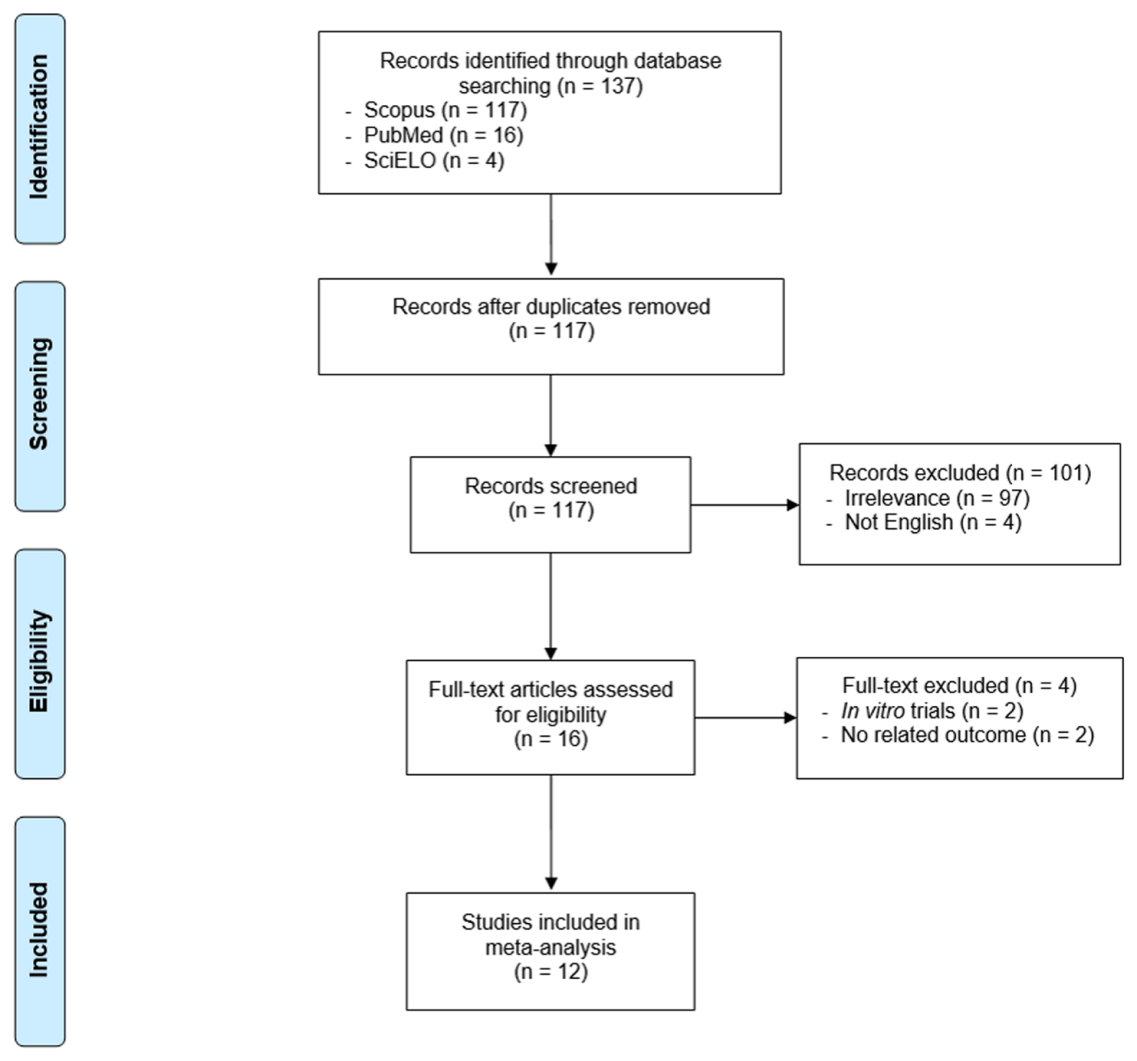
PRISMA flow diagram.

Figure 1 shows the PRISMA flow diagram. A total of 137 records were identified through database searching. Of these, 12 studies were eligible for the current meta-analysis. The essential oil sources included oregano ^11, 22– 24^, thyme ^25, 26^, chavil ^27^, juniper ^7, 28^, and mixed product ^9, 29^. Unfortunately, one study did not define the source of essential oil ^8^. The main characteristics of the included studies are shown in Table 3. Extracted data of outcome measures is available as *Extended data* ^30^.

**Table 3.** Main characteristics of the studies included in the meta-analysis.

Authors	Species	n	Type of ration	EO source	Experimental design	Response variables
Aydin *et al*. ^24^	Sheep	18	Pasture grass + CF	Oregano	CRD	DMI, ADG, FCR
Ribeiro *et al*. ^26^	Sheep	40	TMR	Thyme	RBD	DMI, ADG
Lei *et al*. ^8^	Goats	45	TMR	NI	CRD	ADG
Parvar *et al*. ^27^	Sheep	40	TMR	Chavil	CRD	DMI, ADG, FCR
Canbolat *et al*. ^11^	Sheep	40	TMR	Oregano	CRD	DMI, ADG, FCR
Yesilbag *et al*. ^28^	Goats	18	TMR	Juniper	CRD	ADG
Gümüş *et al*. ^23^	Sheep	24	Wheat straw + CF	Oregano	CRD	ADG
Baytok *et al*. ^25^	Sheep	15	Lucerne hay + CF	Thyme	RBD	ADG, FCR
Malekkhahi *et al*. ^9^	Sheep	10	TMR	Mix A	CRD	DMI, FCR
Özdoǧan *et al*. ^29^	Sheep	20	Alfalfa hay + CF	Mix B	RBD	DMI, ADG, FCR
Canbolat and Karabulut ^22^	Sheep	48	TMR	Oregano	FD	ADG
Chaves *et al*. ^7^	Sheep	20	TMR	Juniper	RBD	DMI, ADG, FCR

n: number of experimental animals; CF: concentrate feed; TMR: total mixed ration; EO: essential oil; NI: no information; Mix A: a mixture of thymol, carvacrol, eugenol, limonene, and cinnamaldehyde EO; Mix B: a mixture of thyme leaf, daphne leaf, sage tea leaf, fennel seed, orange cortes, and myrtle leaf EO; CRD: completely randomized design; RBD: randomized block design; FD: factorial design; DMI: dry matter intake; ADG: average daily gain; FCR: feed conversion ratio.

**Figure 2.**
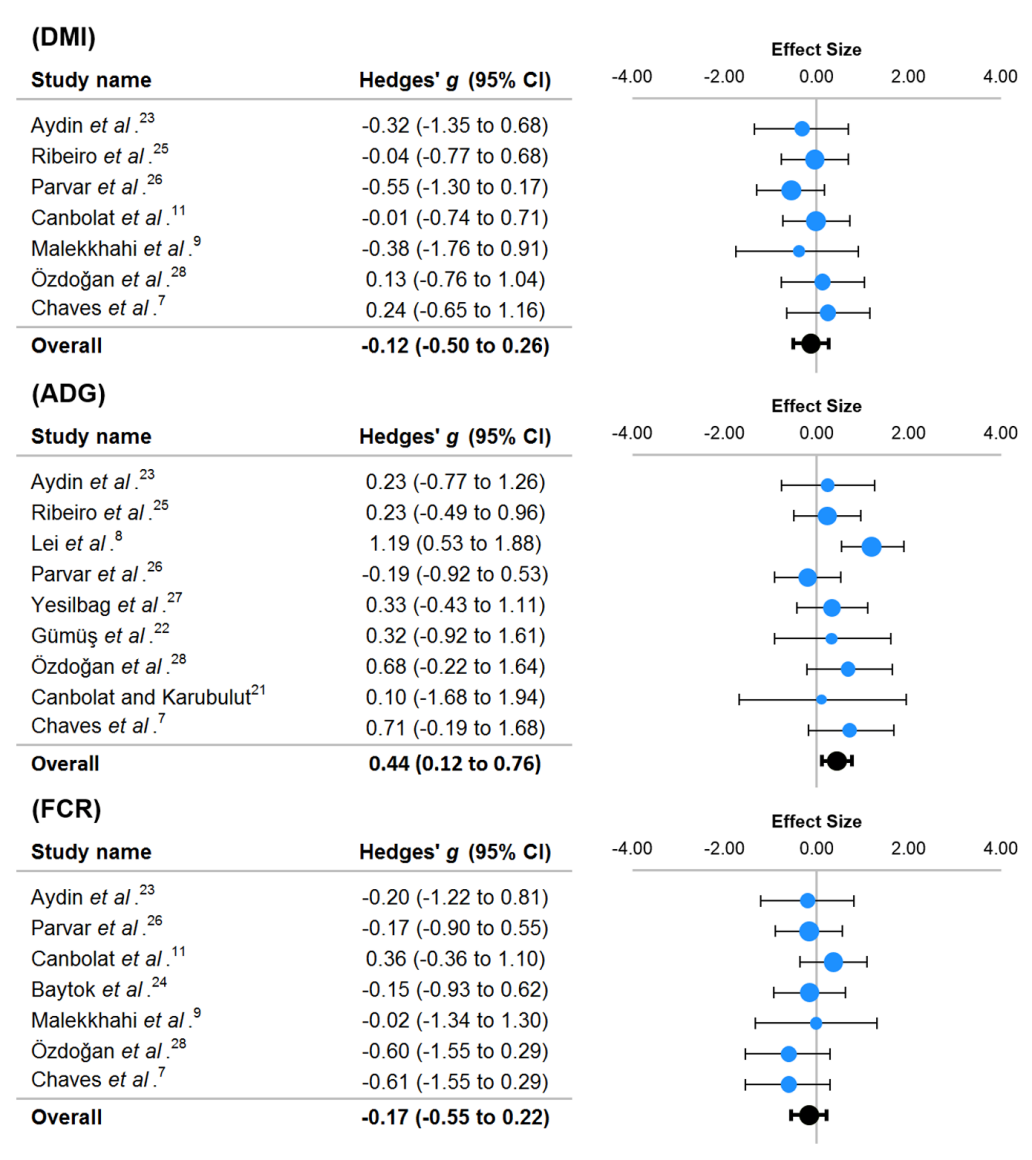
Forest plot of the effect of essential oil supplementation on growth response of small ruminants. DMI: dry matter intake; ADG: average daily gain; FCR: feed conversion ratio.

Data of ADG from two studies ^11, 25^ were considered as outliers because their standardized residual was >|3| and thus were excluded from effect size quantification. Insignificant heterogeneity among studies was detected both for DMI ( *P* of Q = 0.810; I-square = 0.00%), ADG ( *P* of Q = 0.286; I-square = 17.61%), and FCR ( *P* of Q = 0.650; I-square = 0.00%). As can be seen in Figure 2, the overall effect size showed that essential oil supplementation had no significant impact on DMI ( *P* = 0.429) and FCR ( *P* = 0.284), but had a significant positive impact on ADG ( *P* = 0.002). The result of publication bias analysis showed that DMI, ADG, and FCR did not present any significant biases ( *P* >0.10) ( Table 4). The trim and fill method also did not detect any potential missing studies for all parameters.

**Table 4.** Summary of publication bias analysis of the effect of dietary essential oil intervention on growth response of small ruminants.

Parameters	*P* of Begg’s test	*P* of Egger’s test	Missing studies
DMI	0.652	0.879	0
ADG	1.000	0.605	0
FCR	0.652	0.463	0

DMI: dry matter intake; ADG: average daily gain; FCR: feed conversion ratio.

## Discussion

The current meta-analysis showed that dietary essential oils significantly increased ADG of small ruminants. This finding probably related to the antimicrobial activity of essential oils, which could reduce ruminal protozoa population ^31, 32^. Protozoa population may represent up to 50% of the total biomass of rumen microbes ^33^. They have a negative impact on nitrogen utilization by ruminants because they engulf and digest bacteria, thus reducing microbial protein flow to abomasum ^34^. Additionally, the presence of protozoa is also associated with methane production, which is responsible for the loss of up to 12% of gross energy intake by ruminants ^35^. Thereby, the reduction of the ruminal protozoa population by essential oil could increase microbial protein, as well as energy supply, which ultimately could improve the growth rate of small ruminants.

This study provides insight of the potency of essential oil as a growth promoter for small ruminants. However, the current findings should be interpreted with caution due to the limited data available. Moreover, the literature search only covers published literature, which could lead to publication bias. For that reason, further research in this topic is highly encouraged to provide stronger evidence.

## Conclusions

The current meta-analysis reveals that dietary essential oil could improve ADG of small ruminants, without any alteration on DMI and FCR. However, further research in this topic is still highly recommended to provide more robust evidence.

## Data availability

### Underlying data

All data underlying the results are available as part of the article and no additional source data are required.

### Extended data

Figshare: Extended data for ‘The use of essential oils as a growth promoter for small ruminants: a systematic review and meta-analysis’. https://doi.org/10.6084/m9.figshare.12298913.v3 ^30^.

This project contains extracted data of outcome measures (dry matter intake, average daily gain, and feed conversion ratio).

### Reporting guidelines

Figshare: PRISMA checklist for ‘The use of essential oils as a growth promoter for small ruminants: a systematic review and meta-analysis’. https://doi.org/10.6084/m9.figshare.12298034.v2 ^13^.

Data are available under the terms of the Creative Commons Zero "No rights reserved" data waiver (CC0 1.0 Public domain dedication).
