# Supplementary material for: The use of essential oils as a growth promoter for small ruminants: a systematic review and meta-analysis
Source: F1000Res. 2020 Aug 28;9:486. Originally published 2020 Jun 1. [Version 2] doi: 10.12688/f1000research.24123.2 (PMC7331101; doi:10.12688/f1000research.24123.2)
Supplement: Peer review history

# Peer review: Reviewer response for version 1

**DOI:** 10.5256/f1000research.26610.r64168

**Published:** 2020-07-01

**Authors:** Liang Chou Hsia

**Affiliations:** 1 Department of Tropical Agriculture and International Cooperation, National Pingtung University of Science and Technology, Pingtung, Taiwan

The main purpose of this paper tries to investigate whether essential oils have any positive or negative effects on growth performance, such as dry matter intake (DMI), average daily gain (ADG), and feed conversion ratio (FCR). The results shown that the essential oils provided for sheep and goats in 12 studies. Most experiments shown the essential oils can improve the above performance. The present study used the Begg's and Egger's tests which were meta-analysis procedures. Please indicate the original words and then can put abbreviations. It is not necessary to point out the authors' duties in study selection and data extraction. The authors need to make clarification from the literature on why the essential oils can be used as a growth promoter for small ruminants.

Are the rationale for, and objectives of, the Systematic Review clearly stated?

Yes

Is the statistical analysis and its interpretation appropriate?

Yes

Are sufficient details of the methods and analysis provided to allow replication by others?

Yes

Are the conclusions drawn adequately supported by the results presented in the review?

Yes

Reviewer Expertise:

Agricultural production; Aquaculture production; Ecologic system; Conservation; Rural education and extension; Reproduction of animal (ex AI for animals); Nutrition on poultry, pigs, cattle, sheep, goat, dog and cat, etc.; Management for animal production; Animal house design and arrangement; Animal behavior and welfare; Feed processing; Animal waste management; Extension education.

I confirm that I have read this submission and believe that I have an appropriate level of expertise to confirm that it is of an acceptable scientific standard.

# Response

**Published:** 2020-08-20

**Authors:** Faizal Andri

**Affiliations:** Gadjah Mada University, Indonesia

We would like to thank the reviewer for his helpful comments and suggestions. Please see our responses and changes as detailed below.

*1. Please indicate the original words and then can put abbreviations.*

 We have made sure that the original words have indicated before using abbreviations.

*2.  It is not necessary to point out the authors' duties in study selection and data extraction.*

 We have deleted the statement regarding the authors’ duties (see Study Selection and Data Extraction sections).

*3. The authors need to make clarification from the literature on why the essential oils can be used as a growth promoter for small ruminants.*

 We have included the clarification about why essential oils can be used as a growth promoter for small ruminants (see Discussion section).

# Peer review: Reviewer response for version 1

**DOI:** 10.5256/f1000research.26610.r64169

**Published:** 2020-06-17

**Authors:** Linde du Toit

**Affiliations:** 1 Department of Animal and Wildlife Sciences, University of Pretoria, Pretoria, South Africa

In this article the researchers conducted a meta analysis on the effects of essential oils on production parameters of small ruminants. 

 This is a well written article. The objective and methods employed are suitable and clearly defined within the text. The authors could have included the experimental design in the selection criteria as well s the type of rations used in the various studies. The researchers could have broadened the criteria to include more studies in the Meta analysis. Less than 20 studies were included but the authors did discuss this and the need for further research in their discussion. The quality of the article is acceptable for indexing.

Are the rationale for, and objectives of, the Systematic Review clearly stated?

Yes

Is the statistical analysis and its interpretation appropriate?

Yes

Are sufficient details of the methods and analysis provided to allow replication by others?

Yes

Are the conclusions drawn adequately supported by the results presented in the review?

Yes

Reviewer Expertise:

Small ruminant nutrition and livestock GHG emissions

I confirm that I have read this submission and believe that I have an appropriate level of expertise to confirm that it is of an acceptable scientific standard.

# Response

**Published:** 2020-08-20

**Authors:** Faizal Andri

**Affiliations:** Gadjah Mada University, Indonesia

We are grateful to the reviewer for his valuable comments and suggestions. Please see our responses and changes as detailed below.

*1. The authors could have included the experimental design in the selection criteria as well as the type of rations used in the various studies.*

 The use of experimental design and type of ration as selection criteria will left only a small number of eligible studies for synthesis, therefore we do not consider these elements as selection criteria. However, we showed these information as additional study characteristics (see Table 3).

*2. The researchers could have broadened the criteria to include more studies in the Meta-analysis. Less than 20 studies were included but the authors did discuss this and the need for further research in their discussion.*

 The studies in this aspect is currently still very limited and we have addressed this issue (see Discussion section).
